# Challenge to promote change: both young and older adults benefit from contextual interference

**DOI:** 10.3389/fnagi.2015.00157

**Published:** 2015-08-11

**Authors:** Lisa Pauwels, Kathleen Vancleef, Stephan P. Swinnen, Iseult A. M. Beets

**Affiliations:** ^1^Movement Control and Neuroplasticity Research Group, Biomedical Sciences Group, Department of Kinesiology, KU LeuvenLeuven, Belgium; ^2^Leuven Research Institute for Neuroscience & Disease, KU LeuvenLeuven, Belgium

**Keywords:** contextual interference, aging, motor learning, bimanual coordination, skill persistence

## Abstract

Current society has to deal with major challenges related to our constantly increasing population of older adults. Since, motor performance generally deteriorates at older age, research investigating the effects of different types of training on motor improvement is particularly important. Here, we tested the effects of contextual interference (CI) while learning a bimanual coordination task in both young and older subjects. Both age groups acquired a low and high complexity task variant following either a blocked or random practice schedule. Typical CI effects, i.e., better overall performance during acquisition but detrimental effects during retention for the blocked compared with the random groups, were found for the low complexity task variant in both age groups. With respect to the high complexity task variant, no retention differences between both practice schedules were found. However, following random practice, better skill persistence (i.e., from end of acquisition to retention) over a 1 week time interval was observed for both task complexity variants and in both age groups. The current study provides clear evidence that the effects of different practice schedules on learning a complex bimanual task are not modulated by age.

## Introduction

Motor learning, which is defined as a set of processes associated with practice or experience leading to relatively permanent changes in the capability for movement, is very important in every stage of life (Schmidt, 1988). Almost every motor skill used in daily life, such as writing, cycling, walking, etc., needs to be practiced before it can be performed flawlessly. As such, how we practice is highly important for learning and retention. Especially for older adults who may exhibit reduced performance levels (Swinnen et al., 1998; Voelcker-Rehage, 2008; Serbruyns et al., 2015), training may be an optimal tool to learn or to (partly) regain these skills (Voelcker-Rehage, 2008; Seidler et al., 2010). However, to what extent skills can be learned or regained in normal aging by means of training is dependent on several factors, such as task structure, task complexity, task difficulty, and familiarity (Voelcker-Rehage, 2008). Therefore, the impact of training interventions on performance gains in older adults is still ambiguous (Voelcker-Rehage, 2008). While some studies reported greater performance gains for younger compared with older adults (Swinnen et al., 1998; Shea et al., 2006), others reported similar learning gains between both age groups (Carnahan et al., 1993, 1996; Spirduso et al., 1993; Van Dijk et al., 2007) or even the other way around with greater performance improvements for older compared with younger adults (Anshel, 1978). Nevertheless, irrespective of the extent to which performance improves relative to young adults, older adults are capable of achieving reasonable levels of performance gains after a training intervention (Voelcker-Rehage, 2008). In our aging society however, it is crucial to know which types of training lead to the best possible outcome.

With respect to different types of training, Bjork (1994) proposed that adding difficulties into practice will provide more challenges to the learner and this may slow down the learning process. Incorporating conditions into practice which seemingly hamper performance may sound counter-intuitive; however, it is desirable because it may enhance learning outcomes and long-term retention (Bjork, 1994). This was coined with the term “desirable difficulties” (Bjork, 1994). One example of desirable difficulties is the contextual interference (CI) effect in which the practice structure is manipulated by presenting trials in a blocked or random order. In this respect, the CI effect is defined as the learning benefit resulting from practicing various task variants in a random rather than a blocked practice order (Magill, 2011). So far, behavioral research aiming at unraveling the CI effect has had a long history. Back in the 60's, Battig (1966, 1972, 1979) introduced the CI effect in the verbal learning domain. Soon after, the CI effect was demonstrated in the motor skill learning domain, in which Shea and Morgan (1979) demonstrated better retention and transfer performance after learning a three arm-movement task, following a randomized (high CI) compared to a blocked (low CI) practice schedule. However, blocked practice facilitated acquisition performance while performance during random practice was lower (Shea and Morgan, 1979).

Overall, the extensive amount of literature has revealed that the CI effect is a quite robust phenomenon during simple task learning (Magill and Hall, 1990; Magill, 2011). However, despite numerous replications of the CI effect in simple task learning, results in complex task contexts are more equivocal. Wulf and Shea (2002) questioned the generalizability of practice manipulations (e.g., the CI effect) from simple to complex skill learning and concluded that the CI effect led to mixed results in complex skill learning. Whereas some studies succeeded in demonstrating clear benefits of a random practice schedule in complex skill learning (Wrisberg and Liu, 1991; Smith and Davies, 1995; Tsutsui et al., 1998; Maslovat et al., 2004), others did not (Hebert et al., 1996; Jarus and Gutman, 2001). Nevertheless, Pauwels et al. (2014) concluded that although absolute retention differences between blocked and random practice were absent in the most difficult task variant, random practice led to better skill persistence (from the end of acquisition to retention 1 week later) in all task variants. This finding highlights the impact of practice structures on different memory processes (encoding, consolidation and retrieval; (Kantak and Winstein, 2012; Pauwels et al., 2014)). Until recently, research examining the CI effect in older adults has been scarce. Nevertheless, the CI effect has been confirmed in both younger and older adults who learned a serial reaction time task, whereby older adults were equally able to retain the learned sequences to a higher degree after random compared to blocked practice (Lin et al., 2010, 2012).

Little is known about the effects of different practice structures in older adults. Moreover, research investigating the effects of practice structure on motor tasks mainly focused on relatively simple (unilimb) tasks. However, the majority of everyday tasks are more complex and many require a certain degree of coordination between our hands, such as dressing oneself, tying shoelaces, eating, car driving, etc. To the best of our knowledge, there are no studies examining the CI effect in complex (bimanual) task learning in older adults. In this respect, the key question is whether learning a complex task under a random practice schedule is still beneficial in older age. To test this, we compared younger and older adults who completed either a blocked or a random practice schedule. In addition, task complexity varied within each CI condition by presenting three frequency ratios of various complexity levels (Pauwels et al., 2014), which were further categorized according to isofrequency (low complexity task variant) and non-isofrequency (high complexity task variant) ratio types. Based on the previously described literature, we hypothesized typical CI effects, i.e., detrimental effects during acquisition but better retention performance after random practice, in the low complexity (isofrequency) task variant for both young (Magill and Hall, 1990; Wulf and Shea, 2002) and older adults (Lin et al., 2010, 2012). However, with respect to the high complexity (non-isofrequency) task variant, typical CI effects during retention might be absent in both young and older adults, as the effects of CI in complex task learning are still equivocal (Albaret and Thon, 1998; Wulf and Shea, 2002; Guadagnoli and Lee, 2004). The detrimental effects of random practice during acquisition were expected to be more pronounced in older adults because of the increased effort needed during acquisition (Lin et al., 2010, 2012). Finally, as it is known that random practice leads to better skill persistence from the end of acquisition to retention, we hypothesized better skill persistence in both the low and high complexity task variant after random practice for both young (Lin et al., 2010; Pauwels et al., 2014) and older adults (Lin et al., 2010).

## Materials and methods

### Subjects

A total of 96 subjects, of which 48 younger adults (YA; mean age = 19.8 ± 2.2 years; range 18–27 years) and 48 older adults (OA; mean age = 67.3 ± 4.9 years; range 60–80 years), took part in the experiment. All subjects were right-handed as determined by the Oldfield Handedness scale (Oldfield, 1971). Within each age group, subjects were randomly assigned to either of two CI practice conditions: (1) blocked practice and (2) random practice. As such, four different groups were tested; YA-blocked, YA-random, OA-blocked, and OA-random. Detailed group information can be found in Table 1. Within each age group, there were no between-condition differences with respect to age [*p* = 0.585 and *p* = 0.436 for the YA and OA groups, respectively] and laterality quotient [*p* = 0.386 and *p* = 0.434 for the YA and OA groups, respectively]. In addition, the Montreal Cognitive Assessment (MoCA) test was conducted on OA to test for mild cognitive impairment. A standard cutoff score of 26 was used. MoCA scores of the OA-blocked group did not significantly differ with OA-random (*p* = 0.17). Subjects were blind to the purpose of the experiment. Prior to testing, written informed consent was obtained from each subject. The protocol was approved by the local ethical committee of KU Leuven, Belgium, and was in accordance with the Declaration of Helsinki (1964).

**Table 1:** **Group information**.

**Group**	**Amount of subjects (*n*)**	**Mean ± *SD***		
		**Age**	**Laterality**	**MoCA**
YA-blocked	25 (13 female)	19.6±2.2	79.6±23.9	∕
YA-random	23 (11 female)	20±2.3	74.3±16.7	∕
OA-blocked	24 (10 female)	67.9±5.2	92.9±12	27.5±1.3
OA-random	24 (11 female)	66.8±4.7	89.2±20	28.1±1.3

### Instrumentation and task description

**Figure 1:**
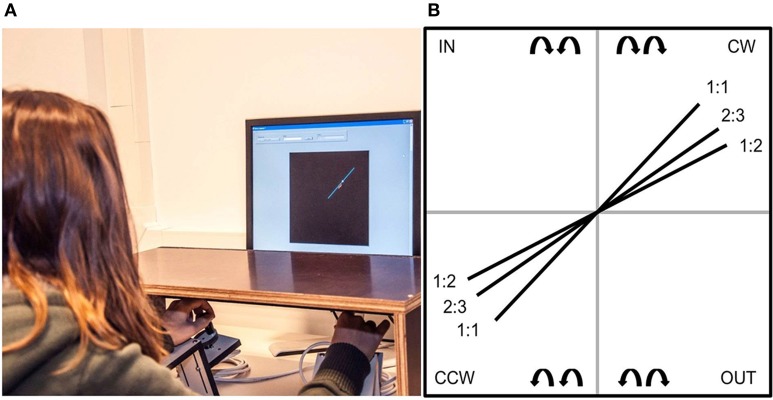
**(A)** *Task set-up*. Subjects were seated in front of a computer screen on which the task was displayed. The response apparatus consisted of two dials which were fixated on a ramp. Direct vision of the forearms was prevented by a horizontal table-top bench. **(B)** *Frequency ratios and coordination directions*. Schematic drawing of the target lines shown on the screen, from which subjects could deduct the three frequency ratios (1:1, 2:3, and 1:2) and coordination directions [clockwise (CW) and counterclockwise (CCW)]. The coordination directions inwards (IN) and outwards (OUT) are shown here, but were not part of the training protocol (Pauwels et al., 2014).

The instrumentation and task was identical to the materials and methods used in Pauwels et al. (2014). A PC-based visuo-motor bimanual tracking task was used. Subjects were seated in front of a computer screen with both lower arms resting on two custom-made adjustable ramps (Figure 1A). The ramps were covered with foam to assure maximal comfort and to minimize fatigue. A dial, consisting of a flat disc (diameter 5 cm) with a vertical peg, was attached at the end of each ramp. The aim of the task was to follow a white target dot along a blue target line on the screen. To perform the required movement, subjects rotated both dials simultaneously by holding each peg between the thumb and index finger. Direct vision of hands and forearms was prevented by placing a horizontal table-top bench over the forearms of the subject. High precision shaft encoders were aligned with the axis of rotation of the dials to record angular displacement (Avago Technologies, sampling frequency = 100 Hz, accuracy = 0.089°). A red cursor showed the current position so that the deviation from the target dot could be corrected. The left dial controlled the vertical component of the red cursor, such that when turning it clockwise, the cursor moved up and when turning it counterclockwise, the cursor moved down. The right dial controlled the horizontal component of the red cursor, such that when turning the dial clockwise or counterclockwise, the cursor moved right and left, respectively. The gain was set to 10 units per rotation, so that 16 complete rotations of both hands were required to complete the target line that consisted of 160 arbitrary units.

A blue target line indicated the main *coordination directions*: both hands could rotate clockwise (CW), counterclockwise (CCW), inwards (IN), and outwards (OUT; Figure 1B). The latter two coordination directions were not used in the current training protocol; however, they were used for instruction prior to testing in order to maximize understanding of the rules of the task (see below). Each coordination direction could be performed at different *frequency ratios*, which was visualized by the slope of the target line (Figure 1B). A target line with a 45° slope indicated a 1:1 frequency ratio, whereby both hands were required to rotate at equal speeds. We used the convention of referring to the left hand first and the right hand second, i.e., L:R. For example, a 1:2 frequency ratio required the right hand to move twice as fast as the left hand.

**Figure 2:**
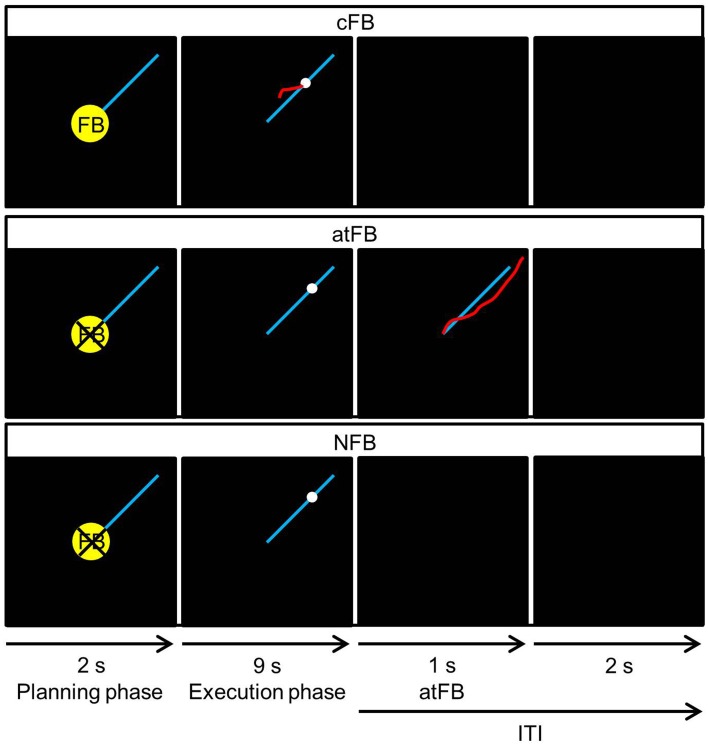
**Three types of FB conditions**. Concurrent visual feedback, provided by a red cursor indicative of subjects' current position, was only provided in the concurrent visual feedback (cFB) condition. In the after-trial feedback (atFB) condition, a motionless representation of the produced red line was provided after the execution phase while no concurrent feedback was provided during the execution phase. In the no feedback (NFB) condition, no concurrent or after-trial feedback was provided. Every trial started with a planning phase of 2 s where a yellow cue, which indicated whether cFB would be given in the upcoming trial, was presented. During the execution phase, the white target dot moved with constant speed along the blue target line for 9 s. In each condition, the inter-trial interval (ITI), i.e., the time between each trial where no movement was required, lasted 3 s. During ITI, atFB was provided for 1 s in the atFB condition. Instead, a black screen was presented in the cFB and NFB condition (Pauwels et al., 2014).

Three types of *feedback conditions* were used: concurrent visual feedback (cFB), after-trial feedback (atFB), and no feedback (NFB; Figure 2). For a more detailed description of the feedback conditions, see Supplementary Material.

### Study design

**Figure 3:**
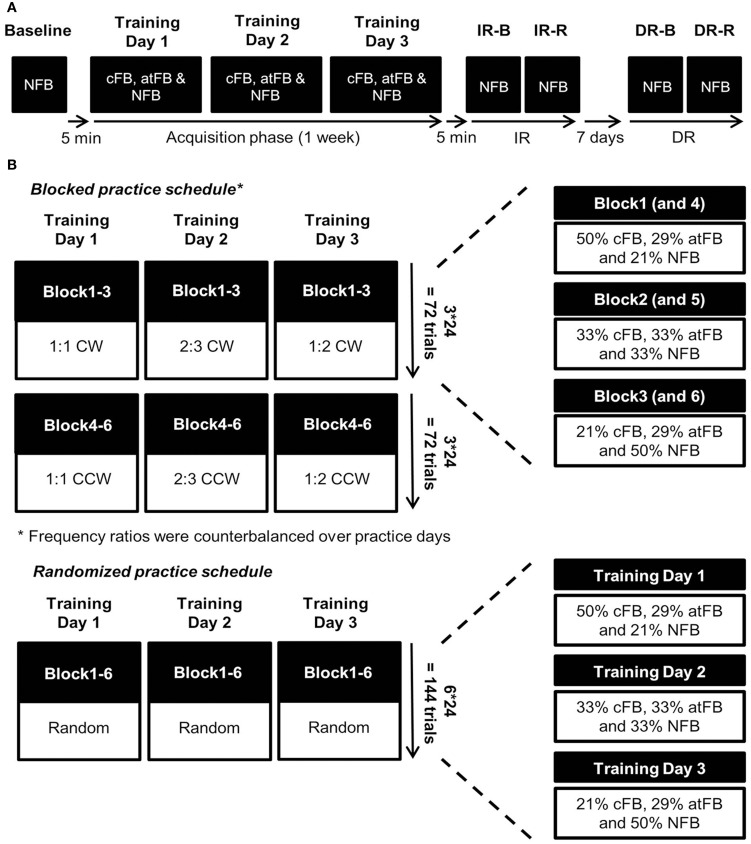
**(A)** *Training protocol*. Baseline performance was assessed without any feedback (NFB) on day 1 prior to training. The acquisition phase consisted of three training days within 1 week. Because of the fading feedback schedule, all three feedback conditions were present during each day of training. Immediate retention (IR) was conducted 5 min after the end of training day 3 and delayed retention (DR) was conducted 7 days later. Both IR and DR consisted of two types of retention schedule, i.e., a blocked (IR-B and DR-B) and a random (IR-R and DR-R) schedule. **(B)** *Blocked and random practice schedules*. Subjects following blocked practice were exposed to one frequency ratio in both clockwise (CW; blocks 1–3) and counterclockwise (CCW; blocks 4–6) directions per day. Frequency ratios were counterbalanced over practice days. In contrast, subjects following random practice were exposed to all six trial types (which were randomly presented) during each block, i.e., four trials per trial type in each block during training. The number of different feedback (cFB, atFB, and NFB) trials and the degree of fading feedback within each trial type was identical for both practice schedules. Therefore, concurrent feedback (cFB) during blocked practice faded over blocks 1–3 after which the fading feedback schedule repeated itself during the next three blocks. In contrast, fading feedback was distributed over days within each trial type when following random practice.

The study design used in the current experiment was similar to the one used in Pauwels et al. (2014). Subjects had to learn three different frequency ratios (1:1, 2:3, and 1:2) in two coordination directions (CW, CCW), i.e., six different trial types, over three practice days within 1 week. The six trial types were trained either under a blocked or random practice schedule. Prior to testing, subjects were informed about the basic requirements to perform the task, i.e., knowledge of the different directions and their associated rotations (CW, CCW, IN, and OUT). No information was given on how to produce the different frequency ratios. To assess whether every subject understood the basic requirements of the task, a familiarization block consisting of four trials, i.e., a 1:1 frequency ratio in each coordination direction (CW, CCW, IN, and OUT), was conducted. For an overview of the training protocol, see Figure 3A.

#### Feedback schedule

In order to prevent reliance on feedback and to optimize learning, we made use of a fading feedback schedule (Winstein and Schmidt, 1990; Kovacs and Shea, 2011, Figure 3B). The feedback schedule was identical to the one used in Pauwels et al. (2014). For a more detailed description of the fading feedback schedule, see Supplementary Material. In order to see how performance without visual guidance evolved in both practice groups, i.e., where the subjects had to produce movements primarily based on an internal representation of the movement pattern instead of having the opportunity to make online corrections based on external visual information, only trials without concurrent visual feedback (65% of 432 trials), i.e., atFB and NFB, were used for analyses of acquisition phase data. For baseline and retention tests, only NFB trials were presented to subjects in order to prevent learning during these tests from online visual feedback or after trial feedback.

#### Baseline

To assess baseline performance, i.e., without prior practice of the to-be-trained trial types, subjects had to perform 12 NFB trials, i.e., two trials per trial type. Frequency ratios during baseline were presented in a blocked manner; however, this was counterbalanced across subjects (cfr. six different practice orders used in acquisition phase).

#### Acquisition phase

The acquisition phase took three training days within 1 week. Subjects in the *blocked condition* learned one frequency ratio per day. In this condition, the order of the learned frequency ratios was counterbalanced over practice days following 1 of 6 practice orders on day 1, 2, and 3, respectively: *practice order 1*. 1:1, 2:3, and 1:2; *practice order 2*. 1:2, 2:3, and 1:1; *practice order 3*. 2:3, 1:1, and 1:2; *practice order 4*. 1:2, 1:1, and 2:3; *practice order 5*. 2:3, 1:2, and 1:1; *practice order 6*. 1:1, 1:2, and 2:3. There were four subjects of YA-blocked for each practice order, except for the first practice order, in which there were five subjects of YA-blocked. With respect to OA-blocked, there were four subjects for each practice order, except for practice order 1 and 6, in which there were 5 and 3 subjects of OA-blocked, respectively. Within each practice day, each frequency ratio was first learned in the CW (blocks 1–3) direction before learning the CCW (blocks 4–6) coordination direction. Subjects in the *random condition* were exposed to all six trial types following a randomized order during every block, i.e., four trials per trial type in each block, of each practice day. The number of practice trials for every trial type was equal for both CI conditions. At the end of practice, a total of 432 trials were completed, of which 150 cFB trials and 282 trials without concurrent FB (132 trials atFB and 150 trials NFB). For each trial type, a total of 72 trials were practiced with 25 trials with and 47 trials without concurrent FB (22 trials atFB and 25 trials NFB). Approximately, 45 min were needed to finish six practice blocks (one practice day). For an overview of the acquisition phase, see Figure 3B.

#### Immediate retention (IR)

Immediately (5 min) after the acquisition phase (last day of practice), subjects were involved in an immediate retention (IR) test to assess the practiced frequency ratios. Two retention schedules, i.e., blocked IR (IR-B) and a randomized IR (IR-R), were used in order to ensure acquisition-retention compatibility was similar for both CI conditions. Both the IR-B and IR-R consisted of 24 NFB trials, i.e., four trials per trial type. First, IR-B was administered in order to avoid learning from randomized practice in the blocked practice groups. The order in which frequency ratios appeared was counterbalanced according to 1 of the 6 practice orders mentioned above. Then (following 1 min of rest), IR-R was conducted in which all learned coordination patterns were presented randomly. Both IR-B and IR-R took 6 min to complete.

#### Delayed retention (DR)

A delayed retention (DR) test, which also consisted of a blocked DR (DR-B) and a randomized DR (DR-R), was conducted 7 days after the last day of practice. The two DR tests were exactly the same as the IR tests.

### Dependent measures

Data were recorded and analyzed with Labview (8.5) software (National Instruments, Austin, Texas, USA). The x and y positions of the target dot and the subjects' cursor were sampled at 100 Hz. Offline analysis was carried out using Matlab R2011b and Microsoft Excel 2010. Accuracy was measured by calculating the error rate based on the average track deviation (ATrD). For each trial, the track deviation was measured as the Euclidian distance between the blue target line and the cursor position at each point in time and then averaged. Better performance is thus reflected by lower values of ATrD. As described in Pauwels et al. (2014), CW and CCW movements were mainly used to provide an extra dimension of complexity to the task (as subjects needed to alternate between them). Therefore, we collapsed CW and CCW data within each frequency ratio. In order to simplify task conditions for the analyses, frequency ratios were classified into two subclasses, i.e., isofrequency (1:1 frequency ratio) and non-isofrequency (2:3 and 1:2 frequency ratio) ratios. As such, the isofrequency pattern reflects a low complexity task variant, whereas the non-isofrequency pattern reflects a high complexity task variant (Sisti et al., 2011; Pauwels et al., 2014). For the acquisition phase analyses, data was averaged across every set of 3 data points in time, which resulted in 16 acquisition phase data points (TR1, TR2, …, TR16) for both the isofrequency and non-isofrequency ratios. Next, as Pauwels et al. (2014) demonstrated that retention performance was not influenced by the context in which retention was obtained; we decided to combine IR-B and IR-R to one data point. The same was done for DR-B and DR-R. In addition to the absolute error measurement (ATrD), we also examined the amount of skill loss for each subject in both task variants. Therefore, a percentage forgetting score was obtained for each subject and was defined as the difference between DR and the end of acquisition (EoA: TR16) divided by EoA and multiplied by 100 (Lin et al., 2010). The additive value of calculating a percentage forgetting score is that forgetting scores of both younger and older subjects with blocked or random practice schedules can be compared directly between both task variants. In addition, we can easily compare our results with the results of Lin et al. (2010). Directional error trials (1.3% for YA-blocked; 1.2% for YA-random; 3.3% for OA-blocked, and 4.1% for OA-random), i.e., when 1 or 2 hands rotated in the wrong direction, were replaced by the according group mean + 3 standard deviations (sd) of the according data point. To reduce the positive skew that was present in our data, data were log-transformed (base 10 logarithm). As such, absolute error scores lower than 1 became negative values.

### Statistical analysis

Statistical analyses were conducted using STATISTICA. For all analyses, the critical probability level was set at *p* < 0.05, two-sided. When significant effects were found, *post hoc* analyses were conducted using Tukey HSD. Full model analysis was conducted using a 2 × 2 × 19 × 2 Age (YA, OA) × CI (blocked, random) × Time (Baseline, TR1-16, IR and DR) × Frequency Ratio (isofrequency, non-isofrequency) repeated measures ANOVA. In order to address the magnitude of the effects, partial eta-squared (η^2^_*p*_) was calculated as a measure of effect size.

#### Baseline

In order to assess whether performance differed prior to practice, baseline performance was analyzed using a 2 × 2 × 2 Age (YA, OA) × CI (blocked, random) × Frequency Ratio (isofrequency, non-isofrequency) repeated measures ANOVA.

#### Acquisition phase

Acquisition phase data were analyzed using a 2 × 2 × 16 × 2 Age (YA, OA) × CI (blocked, random) × Time (TR1-16) × Frequency Ratio (isofrequency, non-isofrequency) repeated measures ANOVA.

#### Immediate and delayed retention

To assess retention performance, a 2 × 2 × 2 × 2 Age (YA, OA) × CI (blocked, random) × Retention Day (IR and DR) × Frequency Ratio (isofrequency, non-isofrequency) repeated measures ANOVA was conducted.

#### Skill persistence

We aimed to test whether random practice leads to better skill persistence than blocked practice in both age groups. Planned comparisons of least square means were conducted on the full model [2 × 2 × 19 × 2 Age (YA, OA) × CI (blocked, random) × Time (Baseline, TR1-16, IR and DR) × Frequency Ratio (isofrequency, non-isofrequency) ANOVA] to test the hypothesized differential change in performance, i.e., difference in post-acquisition processes from the end of acquisition to delayed retention between both CI conditions. To this end, the final time point of training, i.e., end of acquisition (EoA: TR16), was selected and compared with DR. The interaction between Time and CI condition was tested within each age group and for the isofrequency and non-isofrequency ratios separately. In order to conduct these partial interaction contrasts, weights were assigned as follows. As the aim was to examine the CI effect within each age group; a weight of 1 was assigned to YA, while a weight of 0 was assigned to OA and vice versa. To assess the factor CI, each CI practice condition was assigned a weight, i.e., 1 for blocked practice and −1 for random practice. As we want to assess skill persistence in both isofrequency and non-isofrequency ratios separately, a weight of 1 was assigned to one frequency ratio type while the other one received a weight of 0. For the repeated measures factor Time, TR16 (weight of 1) was contrasted to DR (weight of −1), other time points received a weight of 0.

#### Percentage forgetting

A 2 × 2 × 2 Age (YA, OA) × CI (blocked, random) × Frequency Ratio (isofrequency, non-isofrequency) repeated measures ANOVA was conducted to test the influence of CI on percentage forgetting from EoA to DR in both age groups.

## Results

The effect of CI on performance in a bimanual coordination task was tested in both young and older adults. Performance differences were tested before practice (baseline), over the course of practice (acquisition phase), and at retention (IR and DR). In order to get a view into post-acquisition processes, planned comparisons were conducted and a percentage forgetting score was calculated for each subject.

### Baseline

**Figure 4:**
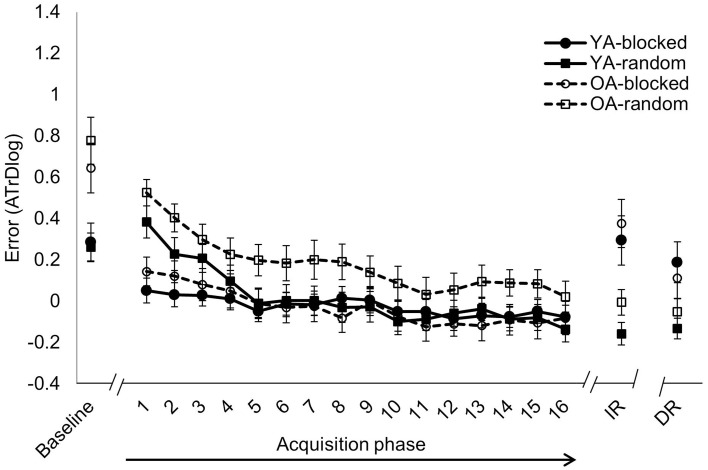
**Isofrequency ratio**. Error score (ATrD, i.e., the log-transformed average track deviation) for baseline, acquisition phase (TR1-16), immediate retention (IR) and delayed retention (DR; mean ± standard error). Within each age group, i.e., younger adults (YA-solid line) and older adults (OA-dashed line), the task was practiced following either a blocked (circles) or random (squares) practice schedule. Better performance is indicated with lower levels of ATrDlog. Performance of OA was significantly lower than performance of YA during baseline (*p* < 0.001, Tukey HSD) but not during acquisition and retention test. Random practice resulted in inferior performance during the acquisition phase (*p* < 0.001) but better retention performance (*p* < 0.001, Tukey HSD) compared to blocked practice.

**Figure 5:**
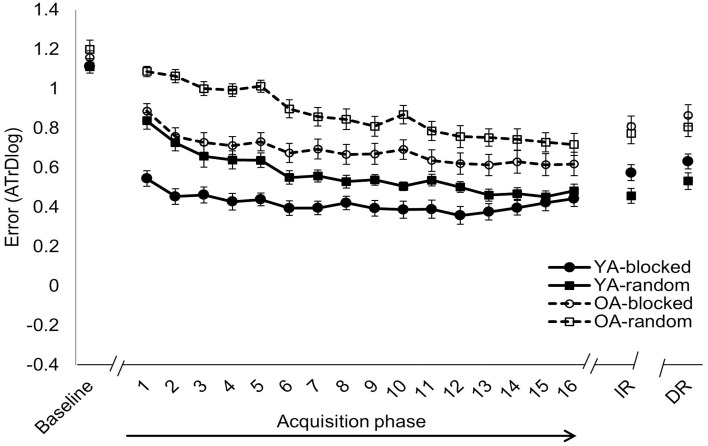
**Non-isofrequency ratios**. Error score (ATrD, i.e., the log-transformed average track deviation) for baseline, acquisition phase (TR1-16), immediate retention (IR) and delayed retention (DR; mean ± standard error). Within each age group, i.e., younger adults (YA-solid line) and older adults (OA-dashed line), the task was practiced following either a blocked (circles) or random (squares) practice schedule. Better performance is indicated with lower levels of ATrDlog. Performance of OA was significantly lower than performance of YA during the acquisition (*p* < 0.001, Tukey HSD) and retention (*p* < 0.001, Tukey HSD) phase. Random practice resulted in inferior performance compared to blocked practice during the acquisition phase (*p* < 0.001). This effect was mainly driven by performance differences at initiation of practice (*p* < 0.001, Tukey HSD).

Results of the 2 × 2 × 2 Age × CI × Frequency Ratio repeated measures ANOVA with respect to baseline measurement are represented in Table 2. A significant main effect of *Age* was observed, indicating that before practice, performance of YA was significantly better than OA. No significant main effect of CI was found which indicated that baseline performance of both CI conditions was comparable. In addition, there was no Age × CI interaction effect indicating that prior to practice, performance within each age group was comparable between both CI conditions. Further, a significant main effect of *Frequency Ratio* was found in which the non-isofrequency pattern was more difficult to perform compared with the isofrequency pattern (Figures 4, 5). Moreover, a significant *Age* × *Frequency Ratio* interaction effect was found in which baseline performance of OA was significantly lower in the isofrequency (*p* < 0.001) but not in the non-isofrequency ratio type (*p* = 0.818) compared with YA (Figures 4, 5). Yet, no CI × Frequency Ratio and Age × CI × Frequency Ratio interaction effects were observed.

**Table 2:** **Baseline**.

**Effect**	***df*_1_, *df*_2_**	***F***	***p***	**η^2^_p_**
Age	1, 92	**18.508**	<**0.001**	**0.167**
CI	1, 92	0.402	= 0.528	0.004
Frequency Ratio	1, 92	**185.156**	<**0.001**	**0.668**
Age × CI	1, 92	0.741	= 0.392	0.008
Age × Frequency Ratio	1, 92	**14.898**	<**0.001**	**0.140**
CI × Frequency Ratio	1, 92	0.121	= 0.729	0.001
Age × CI × Frequency Ratio	1, 92	0.349	= 0.556	0.004

*Results of the 2 = 2 × 2 Age (YA, OA) × CI (blocked, random) × Frequency Ratio (isofrequency, non-isofrequency) repeated measures ANOVA with respect to baseline measurement. Significant effects are indicated in bold*.

### Acquisition phase

Results of the 2 × 2 × 16 × 2 Age × CI × Time × Frequency Ratio repeated measures ANOVA with respect to acquisition data are represented in Table 3. A main effect of *Time* reflecting overall performance improvements over practice was observed. Next, a significant main effect of *Age* was found in which the overall performance of YA was significantly better than performance in OA. There was an *Age* × *Time* interaction effect where OA improved to a greater extent than YA. Next, a significant main effect of *CI* was found in which the overall performance during acquisition was better for the blocked than for the random practice condition (Figures 4, 5). Furthermore, a *CI* × *Time* interaction effect was found in which performance of the blocked condition was significantly better at the beginning of acquisition (TR1; *p* < 0.001) whereas these differences were absent at the end of acquisition (TR16; *p* = 1) as the random condition showed more improvement over the course of training (Figures 4, 5). There were no Age × CI and Age × CI × Time interaction effects indicating that blocked vs. random learning curves were similar in both age groups. Further, a main effect of *Frequency Ratio* was found in which the overall acquisition performance was better for the isofrequency (Figure 4) compared with the non-isofrequency task variant (Figure 5). The *Age* × *Frequency Ratio* interaction effect indicated that OA had disproportionately more difficulty with the non-isofrequency task variant (*p* < 0.001; Figure 5), whereas performance between both age groups was comparable in the isofrequency task variant (*p* = 0.371; Figure 4). The learning curve was very similar between both age groups in both frequency ratio types, as no Age × Time × Frequency Ratio interaction effect was observed. Finally, no interaction effects were observed for CI × Frequency Ratio, CI × Time × Frequency Ratio, Age × CI × Frequency Ratio or Age × CI × Time × Frequency Ratio, indicating that both the overall performance as well as the learning curves of blocked and random practice were independent of frequency ratio and age.

**Table 3:** **Acquisition phase**.

**Effect**	***df*_1_, *df*_2_**	***F***	***p***	**η^2^_*p*_**
Age	1, 92	**22.232**	<**0.001**	**0.195**
CI	1, 92	**14.326**	<**0.001**	**0.135**
Frequency Ratio	1, 92	**390.081**	<**0.001**	**0.809**
Time	15, 1380	**64.873**	<**0.001**	**0.414**
Age × CI	1, 92	1.716	= 0.193	0.018
Age × Frequency Ratio	1, 92	**11.496**	<**0.010**	**0.111**
Age × Time	15, 1380	**1.831**	<**0.050**	**0.020**
CI × Frequency Ratio	1, 92	0.523	= 0.472	0.006
CI × Time	15, 1380	**9.653**	<**0.001**	**0.095**
Age × CI × Time	15, 1380	1.165	= 0.293	0.013
Age × Time × Frequency Ratio	15, 1380	0.479	= 0.952	0.005
CI × Time × Frequency Ratio	15, 1380	1.648	= 0.055	0.018
Age × CI × Frequency Ratio	1, 92	1.082	= 0.301	0.012
Age × CI × Time × Frequency Ratio	15, 1380	0.627	= 0.855	0.007

*Results of the 2 = 2 × 16 = 2 × 2 Age (YA, OA) × CI (blocked, random) × Time (TR1-16) × Frequency Ratio (isofrequency, non-isofrequency) repeated measures ANOVA with respect to acquisition phase data. Significant effects are indicated in bold*.

### Immediate and delayed retention

Results of the 2 × 2 × 2 × 2 Age × CI × Retention Day × Frequency Ratio repeated measures ANOVA for immediate and delayed retention are represented in Table 4. Overall retention performance was better in YA compared with OA, reflected by a main effect of *Age*. Next, the random condition appeared to be beneficial for retention performance compared with the blocked condition as shown by a main effect of *CI* (Figures 4, 5). This was true for both age groups as no Age × CI interaction was observed. Further, no main effect of Retention Day was found, which indicated that performance did not change from IR to DR. This was true within each age group as there was no Age × Retention Day interaction effect. A *CI* × *Retention Day* interaction effect was found in which blocked practice tended to improve from IR to DR, although the *post hoc* test indicated that this effect was not significant (*p* = 0.063), while the random conditions remained stable during this time period (*p* = 0.844). However, performance of blocked and random conditions still significantly differed at DR (*p* = 0.014). No Age × CI × Retention Day interaction effect was observed which indicates that the latter effect was observed in both age groups. Next, a significant main effect of *Frequency Ratio* was found in which retention performance was better for the isofrequency (Figure 4) compared with the non-isofrequency pattern (Figure 5). In line with results of the acquisition phase, age differences were more pronounced in the non-isofrequency pattern, reflected by an *Age* × *Frequency Ratio* interaction effect. Next, a significant *CI* × *Frequency Ratio* interaction effect was found. *Post hoc* tests indicated that the CI effect was only observed in the isofrequency pattern (*p* < 0.001 between blocked and random practice; Figure 4). However, this effect was absent in the non-isofrequency pattern (*p* = 0.677; Figure 5). Moreover, this was confirmed within each age group, as there was no Age × CI × Frequency Ratio interaction effect.

**Table 4:** **Immediate and delayed retention**.

**Effect**	***df*_1_, *df*_2_**	***F***	***p***	**η^2^_*p*_**
Age	1, 92	**11.485**	**<0.010**	**0.111**
CI	1, 92	**18.053**	**<0.001**	**0.164**
Frequency Ratio	1, 92	**194.050**	**<0.001**	**0.678**
Retention Day	1, 92	1.402	=0.240	0.015
Age × CI	1, 92	0.835	=0.363	0.009
Age × Frequency Ratio	1, 92	**5.608**	**<0.050**	**0.057**
Age × Retention Day	1, 92	3.583	=0.062	0.037
CI × Frequency Ratio	1, 92	**8.400**	**<0.010**	**0.083**
CI × Retention Day	1, 92	**5.728**	**<0.050**	**0.059**
Age × CI × Retention Day	1, 92	0.010	=0.753	0.001
Age × CI × Frequency Ratio	1, 92	0.097	=0.757	0.006

*Results of the *2* × *2* × *2* × *2* Age (YA, OA) × CI (blocked, random) × Retention Day (IR and DR) × Frequency Ratio (isofrequency, non-isofrequency) repeated measures ANOVA with respect to immediate and delayed retention data. Significant effects are indicated in bold*.

### Skill persistence

With respect to YA, planned comparisons revealed a significant difference between random and blocked practice from EoA to DR in both isofrequency [*F*_(1, 92)_ = 7.223, *p* = 0.009] and non-isofrequency [*F*_(1, 92)_ = 4.497, *p* = 0.037] ratios. For the OA, the same effect was observed for both frequency ratio task variants [*F*_(1, 92)_ = 7.478, *p* = 0.007 and *F*_(1, 92)_ = 5.897, *p* = 0.017 for isofrequency and non-isofrequency ratios, respectively]. This indicated that performance differentially evolved over the retention interval for each CI practice condition (Figures 4, 5). More specifically, subjects who followed random practice retained their skill to a higher degree compared with subjects who followed blocked practice. *Post hoc* tests indicated that performance remained stable from the end of acquisition to DR after random practice in both age groups. This was true for both frequency ratio task variants (all *p* = 1).

### Percentage forgetting

**Figure 6:**
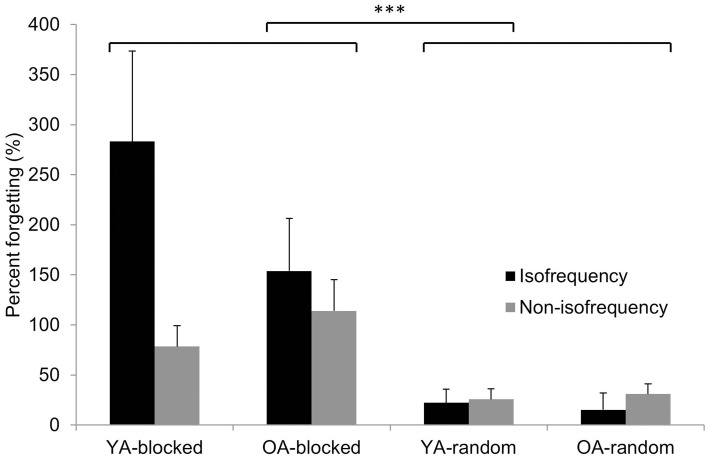
**Percentage forgetting score**. A percentage forgetting score (% forgetting) was obtained for each subject and was defined as the difference between delayed retention (DR) and the end of acquisition (EoA: TR16) divided by EoA and multiplied by 100. Group means + standard errors are displayed for isofrequency (low complexity task variant) and non-isofrequency (high complexity task variant) ratios. Blocked practice resulted in a higher percentage forgetting score compared to random practice (^***^p < 0.001). In addition, blocked practice resulted in higher percentage forgetting in the isofrequency compared with the non-isofrequency ratio (*p* = 0.019, Tukey HSD) while the percentage forgetting score between these two frequency types was not different after random practice (*p* = 0.996, Tukey HSD).

Results of the 2 × 2 × 2 Age × CI × Frequency Ratio repeated measures ANOVA with respect to percentage forgetting score are represented in Table 5. No main effect of Age was observed, indicating that the percentage forgetting score was similar in both age groups. However, a significant main effect of *CI* clearly indicated that random practice resulted in a smaller percentage of forgetting score from EoA to DR (Figure 6). This pattern was evident in both age groups, as no Age × CI interaction effect was observed. As expected, individuals who followed random practice maintained their skill to a higher degree than their blocked practice peers. We did not find a main effect of Frequency Ratio or Age × Frequency Ratio interaction effect. However, a *CI* × *Frequency Ratio* interaction effect was found in which the blocked condition showed more percentage forgetting in the isofrequency compared with the non-isofrequency pattern (*p* = 0.019) while the percentage forgetting score between these two frequency types was not different in the random condition (*p* = 0.996; Figure 6). This was true in both age groups as there was no Age × CI × Frequency Ratio interaction effect.

**Table 5:** **Percentage forgetting**.

**Effect**	***df*_1_, *df*_2_**	***F***	***p***	**η^2^_p_**
Age	1, 92	0.682	= 0.411	0.007
CI	1, 92	**21.413**	<**0.001**	**0.189**
Frequency Ratio	1, 92	3.577	= 0.062	0.037
Age × CI	1, 92	0.636	= 0.427	0.007
Age × Frequency Ratio	1, 92	2.210	= 0.141	0.023
CI × Frequency Ratio	1, 92	**4.916**	<**0.050**	**0.051**
Age × CI × Frequency Ratio	1, 92	1.638	= 0.204	0.017

*Results of the 2 = 2 × 16 = 2 × 2 Age (YA, OA) × CI (blocked, random) × Frequency Ratio (isofrequency, non-isofrequency) repeated measures ANOVA with respect to percentage forgetting data. Significant effects are indicated in bold*.

## Discussion

The aim of this study was to identify whether introducing extra challenges during bimanual coordination practice, such as a random practice schedule, is still desirable at older age. To this end, the CI effect was examined by providing either a random or blocked practice schedule in both younger and older adults in a low (isofrequency pattern) and high (non-isofrequency pattern) complexity task variant. With respect to the acquisition phase, performance during random practice was considerably lower compared with blocked practice. This was confirmed in the young as well as in the older adults for both task variants. An overall pattern was observed in which performance during random practice progressed toward the level of blocked practice. These CI learning curves were similar for both age groups and both task variants. Additionally, we expected that participants who had random practice would outperform the blocked practice group during immediate and delayed retention in the low complexity task variant. In line with our hypothesis, random practice led to better absolute retention in the low complexity task variant, but there were no retention differences between CI conditions in the high complexity task variant. As expected, better skill persistence from the end of acquisition to retention 1 week later was observed for random compared to blocked practice in both task variants. After random practice, percentage forgetting was not influenced by task complexity; but the blocked practice group forgot more in the low than in the high complexity task variant. The effects on absolute retention and percentage forgetting did not interact with age group, suggesting that the effects of CI on post-acquisition memory processes stay stable with age.

### General age differences

As stated in the introduction, motor performance generally deteriorates with age (Swinnen et al., 1998; Voelcker-Rehage, 2008; Serbruyns et al., 2015). This finding is confirmed in the current study as we observed clear baseline differences between younger and older adults. Also during acquisition and retention, the overall performance level of older adults was lower than younger adults. This effect was mainly driven by differences in the more complex, non-isofrequency, task variant. This is in line with existing literature stating that older adults are readily able to reach similar performance levels as younger adults during relatively simple coordination tasks. However, in more complex coordination patterns (or other tasks which require more effortful processing), relative age differences become more apparent (Swinnen et al., 1998; Serrien et al., 2000; Voelcker-Rehage, 2008). Nevertheless, whereas previous work suggested that learning gains in older adults are reduced in more complex tasks (Voelcker-Rehage, 2008), no age-related learning deterioration was observed in the current study.

### Contextual interference and aging

#### Acquisition phase

In line with the existing CI literature, blocked practice facilitated performance during the acquisition phase compared to random practice (Magill and Hall, 1990; Brady, 1998; Lee and Simon, 2004). Nevertheless, as practice progressed, performance during random practice progressed toward blocked practice levels in both age groups and for both levels of task difficulty. The observation of performance differences between blocked and random practice being more apparent in the beginning of practice compared with the end of practice is a well-known concept in CI literature (Shea and Morgan, 1979; Shea et al., 1990; Pauwels et al., 2014). Maslovat et al. (2004) for example found that, when longer practice schedules are provided, subjects following random practice can outperform subjects following blocked practice during bimanual coordination task learning. Remarkably, the different time courses of both CI conditions were not affected by age in the current study. Hence, even in the most complex task variant, the additional difficulty of random practice did not affect learning gains in the older adults. This was not in line with the study of Lin et al. (2010), where there was less improvement for the older adults, but not for the younger adults, for random compared to blocked practice.

#### Retention

Both younger and older adults who followed a random practice schedule outperformed their blocked practice peers during retention in the low complexity task variant. However, this was not true in the high complexity task variant as there were no absolute retention differences between CI conditions in both age groups. These findings are consistent with the results of Pauwels et al. (2014); however, only younger adults were tested in this study. Lin et al. (2010) observed retention differences favoring random practice after learning a serial reaction time task in both younger and older adults. To the best of our knowledge, no studies have been conducted exploring the CI effect in older adults using more complex task variants. Hence, we can state that increasing the training challenge by introducing a random practice schedule had no beneficial effect on absolute retention performance for both young and older adults in the most complex task variant.

#### Skill persistence and skill forgetting

Random practice led to better skill persistence from the end of acquisition to retention 1 week later in both task variants compared with blocked practice. This effect was already observed in Pauwels et al. (2014) for younger adults and is now confirmed in older adults. However, as already mentioned in the methods section, the percentage forgetting score provides an additive value to our analyses as we can directly compare forgetting scores between younger and older individuals, between both CI practice schedules and between both task variants. With respect to the percentage forgetting score, in line with Lin et al. (2010) who used a serial reaction time task, we confirmed that blocked practice led to more skill forgetting from the end of acquisition to retention 1 week later, regardless of age. Hence, post-acquisition processes, which provide a basis for long-term memory, do not appear to be impaired by age (Lin et al., 2010). An interesting new finding was that (again regardless of age) blocked practice induced higher percentage forgetting scores in the low compared with the high complexity task variant whereas forgetting after random practice was similar for both task variants.

#### Desirable difficulties

Did the extra challenge, induced by a random practice schedule, lead to a system overload while learning a complex task variant? At first sight, the answer seems to be positive as absolute retention differences favoring random practice were present in the simplest but not in the most complex task variant. On the other hand, post-acquisition processes after random practice were not affected in the most complex task variant.

According to the *challenge point framework* of Guadagnoli and Lee (2004), the effects of practice conditions (such as CI) are dependent on the skill level of the learner and task complexity. As such, the optimal challenge point represents the optimal amount of challenge (i.e., difficulty induced by different practice conditions) an individual of a specific skill level would need in order to optimize learning. In this respect, the authors state that increasing difficulty by introducing a random practice schedule is recommended when practicing a relatively simple task. In contrast, practicing a more complex task would be sufficiently challenging to the learner, and consequently, adding difficulty would be redundant or even detrimental for the learner (Guadagnoli and Lee, 2004). However, our data suggest that random practice was not redundant, nor detrimental, when learning a more complex task variant as performance remained stable from the end of acquisition to retention on week later. This suggests that the beneficial effects of random practice on post-acquisition processes, even when learning a more demanding task variant, are preserved. Rather, forgetting after blocked practice was influenced by complexity of task variants. In this respect, it appeared that the extra challenge of the more complex task variant positively influenced post-acquisition processes in subjects who were involved in blocked practice. In other words, the extra challenge of task complexity tended to be a *desirable difficulty* in the blocked group, which might have contributed to the lack of absolute retention differences in the more complex task variant.

## Conclusions

The current study provides strong evidence that the effects of different practice schedules on learning a complex (bimanual) task do not change with age. Despite the fact that the overall performance of older adults is lower, older adults are able to reach considerable performance gains. Moreover, post-acquisition processes after random practice were not influenced by age, even when task demands were higher in the more complex task variant. Furthermore, we provided clear evidence for the benefits of random practice in a relatively simple task variant; however, no retention differences between CI conditions were observed in the more complex task variant. The extra challenge of a more complex task variant tended to be desirable for blocked practice, eliminating retention differences between blocked and random practice.

## Author contributions

Conceived and designed the experiments: LP, KV, SS, and IB. Performed the experiments: LP. Analyzed the data: LP, KV, and IB. Contributed materials/analysis tools: LP, KV, SS, and IB. Wrote the paper: LP, KV, SS, and IB. All authors approved the final version of the manuscript.

## Funding

LP is funded by an aspirant fellowship of the Research Foundation—Flanders (FWO). The funders had no role in study design, data collection and analysis, decision to publish, or preparation of the manuscript. This work was supported by a grant from the Research Program of the Research Foundation—Flanders (Fonds Wetenschappelijk Onderzoek—FWO; http://www.fwo.be/) (G0721.12, G0708.14), from the Research Fund of KU Leuven, Belgium (OT/11/071), and Grant P7/11 from the Interuniversity Attraction Poles program of the Belgian federal government.

### Conflict of interest statement

The authors declare that the research was conducted in the absence of any commercial or financial relationships that could be construed as a potential conflict of interest.
