# Supplementary material for: Challenge to promote change: both young and older adults benefit from contextual interference
Source: Front Aging Neurosci. 2015 Aug 11;7:157. doi: 10.3389/fnagi.2015.00157 (PMC4531253; doi:10.3389/fnagi.2015.00157)
Supplement: Supplementary file 1 [file DataSheet1.DOCX]

***Supplementary Material***

**Challenge to promote change: both young and older adults benefit from contextual interference**

**Lisa Pauwels^1^*, Kathleen Vancleef^1^, Stephan P. Swinnen^1, 2^ and Iseult A.M. Beets^1^**

^1^Movement Control and Neuroplasticity Research Group, Biomedical Sciences Group, Department of Kinesiology, KU Leuven, Leuven, Belgium

^2^Leuven Research Institute for Neuroscience & Disease (LIND), KU Leuven, Leuven, Belgium

*** Correspondence:** Lisa Pauwels, Movement Control and Neuroplasticity Research Group, Biomedical Sciences Group, Department of Kinesiology, KU Leuven, Tervuursevest 101, Leuven, 3001, Belgium.

[lisa.pauwels@faber.kuleuven.be](mailto:lisa.pauwels@faber.kuleuven.be)

# Supplementary Materials and Methods

## Types of feedback conditions

Three types of *feedback conditions* were used: concurrent visual feedback (cFB), after-trial feedback (atFB) and no feedback (NFB) (Figure 2). In all conditions, the blue target line and the white target dot were presented. In each trial, the white target dot was first covered by a yellow cue which indicated whether cFB would be given in the upcoming trial. The cue and target dot remained motionless in the center of the screen for 2 s. No movement was required, but the subject was instructed to plan the movement (planning phase). Then, an auditory cue was provided to indicate the start of the execution phase. The execution phase lasted 9 s. During the execution phase, the white target dot moved with constant speed starting from the center of the display, along the blue target line, towards the periphery. The goal of each trial was to generate the correct track by turning both dials in the proper direction and to control the relative velocity between both hands such that the correct frequency ratio could be produced. The inter-trial interval (ITI) lasted 3 s in which a black screen was presented. In the cFB condition, current performance was visualized online by a red cursor which contained the most recent information of the subjects’ movement track (1 s), upon which movements could be corrected. During atFB trials, no red cursor was shown, but the same blue target line and the white target dot were presented. After-trial FB was provided immediately after each atFB trial by presenting a motionless representation of 1 s consisting of the produced red line, representing the produced movement next to the required target line, indicating the discrepancy between the produced and the required movement. In NFB trials, the blue target line and the moving white target dot were also presented, but neither cFB nor atFB was provided. Thus, in both the atFB and NFB conditions, subjects were required to track the target pathway of the frequency ratio without the guidance of concurrent visual feedback.

## Feedback schedule

In order to prevent reliance on feedback and to optimize learning, we made use of a fading feedback schedule ([Winstein and Schmidt, 1990](#_ENREF_3); [Kovacs and Shea, 2011](#_ENREF_1)). The feedback schedule was identical to the one used in [Pauwels et al. (2014](#_ENREF_2)). That is, for each trial type, we gradually reduced the number of trials in which we provided concurrent visual feedback. There were 72 trials per trial type (i.e. per frequency ratio in either CW or CCW coordination directions), across training for all practice groups. Each practice day consisted of 144 trials with six blocks of 24 trials each (Figure 3B). The numbers of cFB trials were gradually reduced (50% for trial 1-24; 33% for trial 25-48 and 21% for trial 49-72) while the NFB trials gradually increased (21%; 33% and 50% respectively) for each trial type (Figure 3B). The number of atFB trials was kept relatively constant for each trial type throughout training (29% for trial 1-24, 33% for trial 25-48 and 29% for trial 49-72). In the blocked condition, feedback faded from block 1 to 3 after which the fading feedback schedule repeated itself for the next trial types, i.e. during every following 3 practice blocks. In the random condition, all 6 trial types were randomly presented during every block across training. That is, during every training day, all 6 trial types were practiced, i.e. each trial type was presented 4 times during each block, and the trial number of each trial type (i.e. 72) was spread across the three training days. The fading feedback schedule in the random condition was therefore spread over training days for each trial type. Concurrent FB was given for 50 % of trials on day 1, 33 % on day 2, and 21 % on day 3 for all trial types. Within each training day, cFB also generally faded, starting with more cFB trials at the beginning of the training day and ending with more NFB trials. The number of cFB, atFB and NFB trials and the degree of fading within each trial type was identical in both CI conditions.

# References

Kovacs, A.J., and Shea, C.H. (2011). The learning of 90 degrees continuous relative phase with and without lissajous feedback: External and internally generated bimanual coordination. *Acta Psychol. (Amst).* 136**,** 311-320. doi: 10.1016/j.actpsy.2010.12.004

Pauwels, L., Swinnen, S.P., and Beets, I.a.M. (2014). Contextual interference in complex bimanual skill learning leads to better skill persistence. *PloS one* 9**,** e100906. doi: 10.1371/journal.pone.0100906

Winstein, C.J., and Schmidt, R.A. (1990). Reduced frequency of knowledge of results enhances motor skill learning. *J. Exp. Psychol. Learn. Mem. Cogn.* 16**,** 677-691. doi: 10.1037//0278-7393.16.4.677
